# Foveal structure in nanophthalmos and visual acuity

**DOI:** 10.1007/s10792-020-01633-9

**Published:** 2020-11-13

**Authors:** Hideaki Okumichi, Katsumasa Itakura, Yuki Yuasa, Atsuhiko Fukuto, Yoshiaki Kiuchi

**Affiliations:** Department of Ophthalmology and Visual Science, Hiroshima University Graduate School of Biomedical and Health Sciences, 1-2-3 Kasumi, Minamiku, Hiroshima, 7348551 Japan

**Keywords:** Nanophthalmos, Visual acuity, OCT angiography, Foveal avascular zone

## Abstract

**Purpose:**

To evaluate the fovea in nanophthalmic eyes using spectral domain optical coherence tomography (SD-OCT) and OCT angiography (OCTA), and to investigate the relationship between the macular microstructure and visual acuity.

**Methods:**

This is a retrospective case series of five nanophthalmic patients. The foveal avascular zone (FAZ) area was measured in superficial and deep vascular layers with OCTA. The thickness of the inner retinal layer (IRL) was measured with SD-OCT. The ratio of the foveal and parafoveal IRL thickness (fIRL/pIRL ratio) was calculated. The relationship between these parameters and visual acuity was then investigated.

**Results:**

Eight eyes were identified as nanophthalmic with a mean axial length of 17.19 ± 1.44 mm (range: 15.71 to 19.88 mm). The mean best-corrected visual acuity (BCVA) in the logarithm of the minimum angle of resolution (logMAR) was 0.12 ± 0.18 (range: − 0.18 to 0.40). OCTA showed that FAZs were either absent or undeveloped in the superficial and deep capillary plexuses. Two patients did not show any visual impairments despite small FAZ and a shallow foveal depression. Although the BCVA was significantly correlated with the deep FAZ size, it did not correlate with the superficial FAZ size, axial length, or fIRL/pIRL ratio. However, the refractive error, axial length, and deep FAZ size were all significantly correlated with the fIRL/pIRL ratio.

**Conclusions:**

The FAZs were commonly found to be small in the superficial and deep capillary plexuses. Although the deep FAZ size correlated with visual acuity, it is unclear whether the retinal microstructure and the FAZ size are responsible for the visual impairments observed in the same individuals.

## Introduction

Nanophthalmos is a rare congenital eye disorder that is characterized by an axial length under 20 mm. This condition causes a variety of morphological and functional anomalies, such as hyperopia, microcornea, shallow anterior chamber, angle-closure glaucoma, and thickened sclera [1, 2]. This condition is also associated with foveal hypoplasia and an absent foveal avascular zone (FAZ) [3].

Although previous reports have analyzed retinal and choroidal changes occurring in nanophthalmic eyes, using either optical coherence tomography (OCT) [4–6] or fluorescein angiography [3], it remains unclear whether a causal relationship exists between the abnormal retinal vascular structure and a reduction in visual acuity. OCT angiography (OCTA) has been validated as a novel and non-invasive method to measure blood flow within the fundus in two different vascular layers (superficial and deep vascular plexuses) without the need for a fluorescein dye injection [7, 8].

The purpose of this report is to describe the foveal structure and vasculature of nanophthalmic eyes using OCTA and spectral domain (SD)-OCT and to investigate the relationship between these measures and visual acuity.

## Patients and methods

This is a retrospective case series study of five consecutive nanophthalmic patients examined in the glaucoma department of Hiroshima University Hospital, Hiroshima, Japan, from June 1, 2016, to January 31, 2018. All procedures were performed in accordance with the Declaration of Helsinki and were approved by the Ethics Committee of Hiroshima University Hospital (E-1709). Written informed consent was obtained from all patients.

Five participants underwent comprehensive ophthalmic examinations, which included slit-lamp biomicroscopy, gonioscopy, fundus photography, SD-OCT, and OCTA. Measurements of axial length, best-corrected visual acuity (BCVA), and refractive correction were also obtained. A diagnosis of nanophthalmos was given in cases where the axial length was under 20 mm. OCTA and SD-OCT were performed with an RTVue XR Avanti (Optovue Inc., Fremont, CA, USA) imaging system using a 3.00 × 3.00 mm resolution to evaluate both the superficial and deep capillary plexus layers. Two eyes were excluded from further analyses: one had proliferative vitreoretinopathy (Case 4, OS) and the other did not meet the axial length (20.31 mm) criterion for nanophthalmos (Case 5, OD).

In order to evaluate the extent of foveal development, measurements of FAZ size and thickness were obtained for both the fovea and parafovea. The foveal to parafoveal inner retinal layer ratio (fIRL/pIRL ratio) was subsequently calculated as previously described [9]. During early foveal development, retinal axons and blood vessels are displaced outward to form the invagination of the fovea, thus producing the FAZ [10]. Therefore, an increased fIRL/pIRL ratio indicates an immature and underdeveloped fovea [11]. From the OCTA images, the FAZ area was measured manually using Adobe Photoshop (version CC 2017; Adobe Systems Inc., San Jose, CA, USA). The length or area size measured in OCT/OCTA images is needed to be corrected by axial length. The coefficient by axial length (CoAx) was calculated as CoAx = 3.382 [0.01306 (Ax − 1.82)]. The measured area size was corrected by the factor CoAx^2^ [12]. The IRL thickness was also manually measured from the SD-OCT images for both fovea and parafovea (1000 µm nasal from the foveal center) sites. Finally, the fIRL/pIRL ratio was subsequently calculated.

Statistical analyses were performed with JMP® Pro 14.0.0 (SAS Institute Inc., Cary, NC). Correlations between the corrected superficial and deep FAZ sizes, BCVA in the logarithm of the minimum angle of resolution (logMAR), fIRL/pIRL ratios, refractive errors, and axial lengths were investigated using Spearman’s rank correlation coefficient. Statistical significance was set at *P* < 0.05.

## Results

Eight eyes were diagnosed as nanophthalmic. The mean age of the participants was 49 ± 13 years (range: 37–70 years), the mean axial length was 17.19 ± 1.44 mm (range: 15.71–19.88 mm), and the mean BCVA in logMAR was 0.12 ± 0.18 (range: − 0.18–0.40) (Table 1). Gonioscopy examinations showed shallow anterior chambers and narrow angles (Shaffer 2, Sheie 4) in all patients.

**Table 1:** OCTA measurements in five nanophthalmos cases

Case	Age (years)	Gender	OD/OS	BCVA (logMAR)	Refraction (D)	Axial length (mm)	Superficial FAZ area (mm^2^)	Deep FAZ area (mm^2^)	fIRL thickness (μm)	pIRL thickness (μm)	fIRL/pIRL ratio
1	70	Female	OD	0.40	+ 8.00	16.27	0.068	0.147	78.13	208.33	0.38
			OS	0.30	+ 8.00	16.25	0.215	0.413	33.85	187.50	0.18
2	48	Female	OD	0.15	+ 9.75	18.06	0.206	0.259	28.65	210.94	0.14
			OS	0.15	+ 9.75	18.01	Defect	0.423	20.83	169.27	0.12
3	45	Male	OD	0	+ 16.00	15.71	Defect	Defect	177.08	242.19	0.73
			OS	0	+ 16.00	15.83	Defect	Defect	234.38	213.54	1.10
4	43	Female	OD	− 0.18	+ 10.25	17.48	Defect	Defect	226.56	234.38	0.97
5	37	Female	OS	0.10	+ 3.50	19.88	0.123	0.050	7.81	138.02	0.06

*OCTA* optical coherence tomography angiography, *BCVA* best-corrected visual acuity, *logMAR* logarithm of minimum angle of resolution, *D* diopter, *FAZ* foveal avascular zone, *fIRL* foveal inner retinal layer, *pIRL* parafoveal inner retinal layer

**Fig. 1:**
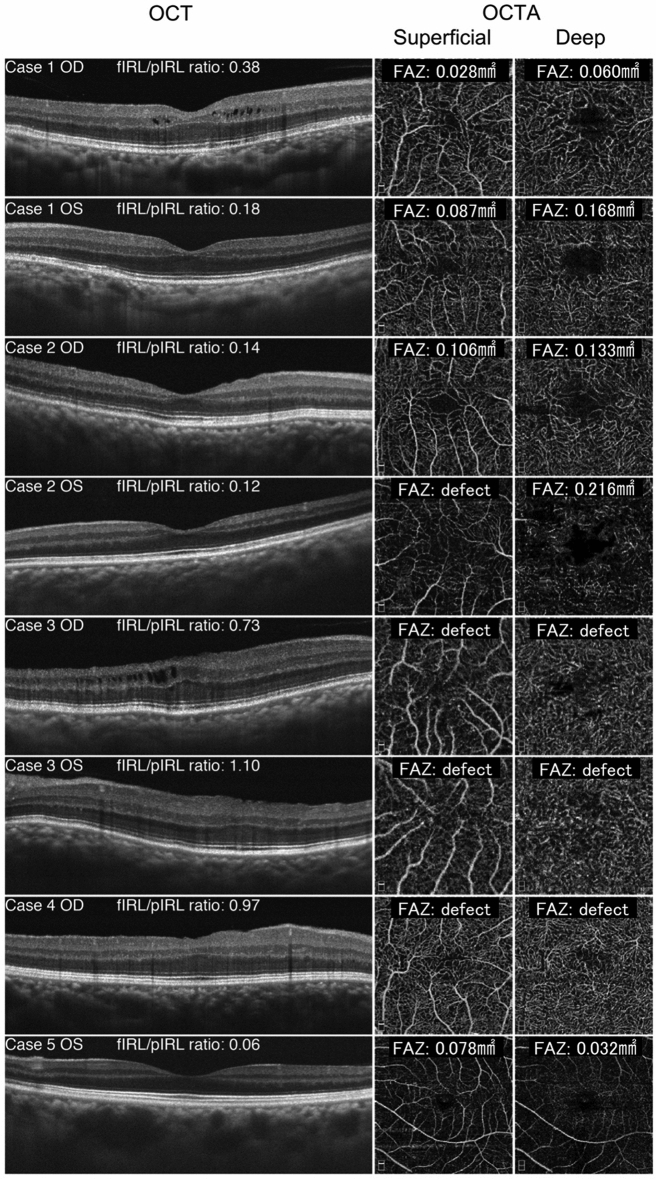
Comparison of the cross-sectional optical coherence tomography (OCT) images (left) and optical coherence tomography angiography (OCTA) images of the superficial (middle) and deep (right) capillary plexuses from all eight eyes. The thickness of the foveal inner retinal layer (fIRL) and parafoveal inner retinal layer (pIRL), in addition to the area of the foveal avascular zone (FAZ), were all measured manually. Subsequently, fIRL/pIRL ratio was calculated and the size of FAZ area was corrected by axial length

Using OCTA, it was ascertained that the FAZ was either absent or rudimentary in both the superficial and deep vascular layers (Table 1) (Fig. 1). The FAZ was completely absent in three eyes (Cases 3 and 4) and was small or underdeveloped in the superficial layer of four eyes (Cases 1, 2, and 5). The corrected size of observable FAZs varied from 0.028 to 0.106 mm^2^ in superficial layers and from 0.032 to 0.216 mm^2^ in deep layers (Table 2). Five eyes (Cases 1, 3, and 4) were found to have shallow foveal pit based on OCT findings. Three eyes (Cases 3 and 4) had normal visual acuity despite having FAZ abnormalities in either the superficial or deep capillary plexuses and an abnormal foveal depression.

**Table 2:** Correction of FAZ area size

Case	OD/OS	Axial length (mm)	CoAx	Corrected superficial FAZ area (mm^2^)	Corrected deep FAZ area (mm^2^)
1	OD	16.27	0.64	0.028	0.060
	OS	16.25	0.64	0.087	0.168
2	OD	18.06	0.72	0.106	0.133
	OS	18.01	0.72	0	0.216
3	OD	15.71	0.61	0	0
	OS	15.83	0.62	0	0
4	OD	17.48	0.69	0	0
5	OS	19.88	0.80	0.078	0.032

*FAZ* foveal avascular, *CoAX* coefficient by axial length

**Fig. 2:**
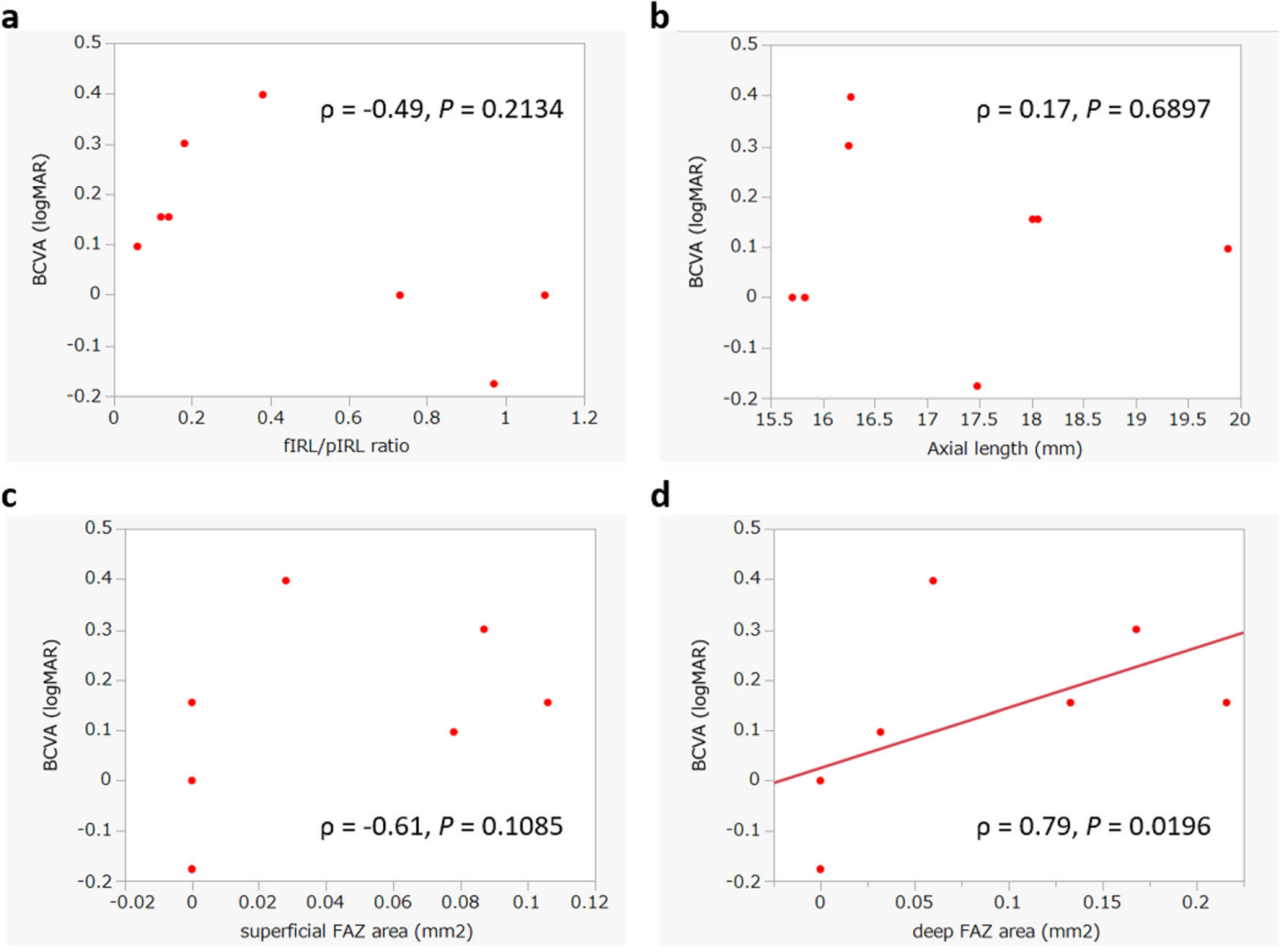
Correlations between the visual acuity (logMAR), the fIRL/pIRL thickness ratio, the axial length, and the size of superficial and deep FAZ. Correlations were computed using Spearman’s rank correlation coefficient. The correlation between **a** the visual acuity and the fIRL/pIRL ratio, between **b** the visual acuity and the axial length and between **c** the visual acuity and the size of superficial FAZ were not significant, whereas the correlation between **d** the visual acuity and the size of deep FAZ (*ρ* = 0.79, *P* = 0.0196) was significant

**Fig. 3:**
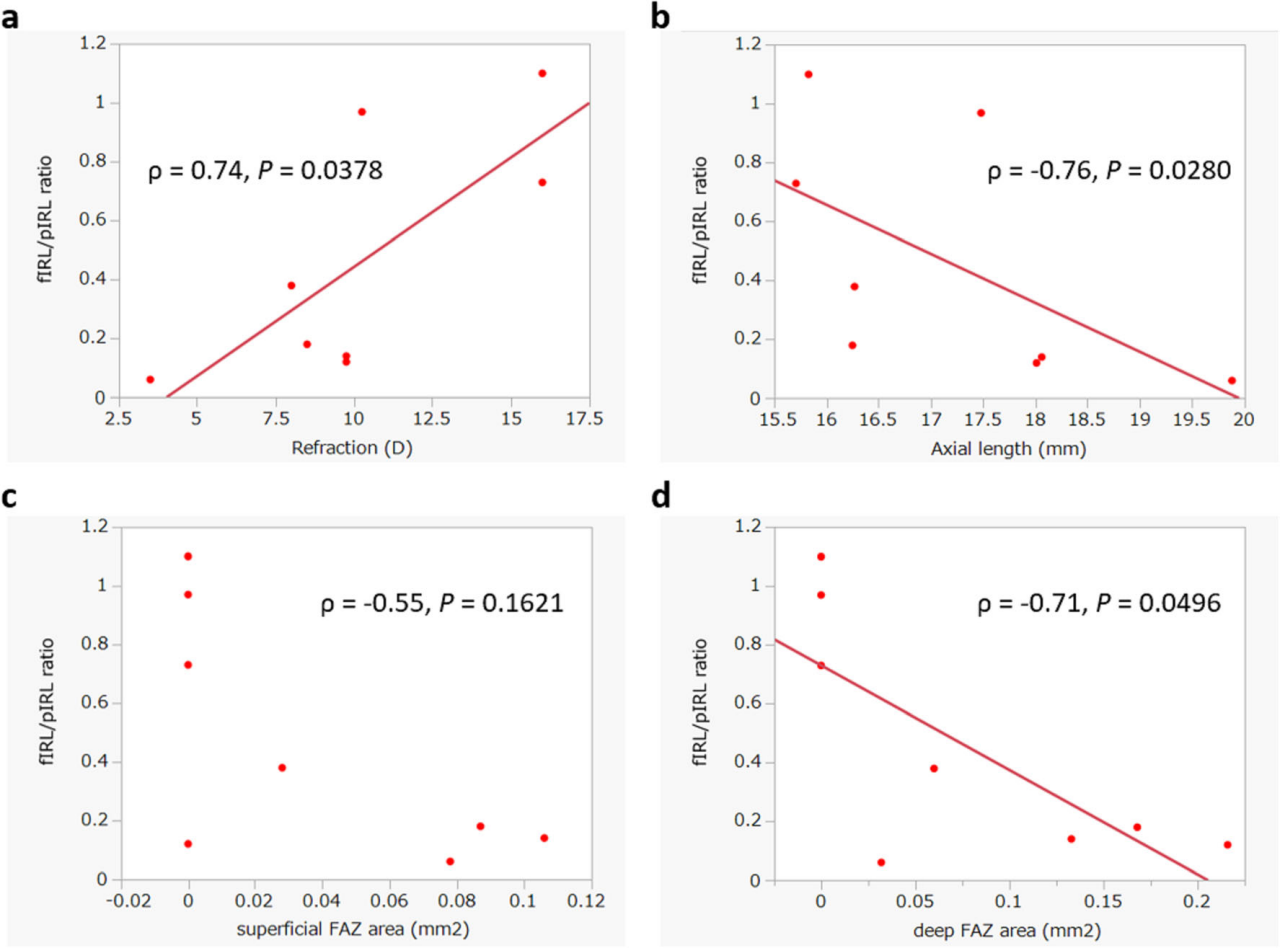
Correlations between the fIRL/pIRL ratio, the refractive error, the axial length, and the size of superficial and deep FAZ. Correlations were computed using Spearman’s rank correlation coefficient. The correlation between **a** the fIRL/pIRL ratio and the refractive error (*ρ* = 0.74, *P* = 0.0378), between **b** the fIRL/pIRL ratio and the axial length (*ρ* = −0.76, *P* = 0.0280) and between **d** the fIRL/pIRL ratio and the size of deep FAZ (*ρ* = −0.71, *P* = 0.0496) were significant, whereas the correlation between **c** the fIRL/pIRL ratio and the size of superficial FAZ was not significant

Correlation analyses revealed a significant relationship between the BCVA in logMAR and the size of the deep FAZ (*ρ* = 0.79, *P* = 0.0196). However, the BCVA did not correlate with the superficial FAZ area, the fIRL/pIRL ratio, refractive error, or axial length (Fig. 2). Although the size of both the superficial and deep FAZ was not correlated with the axial length, a significant correlation was found between the deep FAZ size and the fIRL/pIRL ratio (*ρ* = −0.71, *P* = 0.0496). The fIRL/pIRL ratio was also significantly correlated with the refractive error (*ρ* = 0.74, *P* = 0.0378) and with the axial length (*ρ* = −0.76, *P* = 0.0280), but not with the superficial FAZ size (Fig. 3). The refractive error was not significantly correlated with either the superficial FAZ or deep FAZ size, nor with the axial length. The superficial and deep FAZ sizes were not significantly correlated with one another (Table 3).

**Table 3:** Spearman’s rank correlation coefficient

	Coefficient (*ρ*)	*P*
BCVA vs.		
Superficial FAZ	0.61	0.1085
Deep FAZ	0.79	0.0196*
fIRL/pIRL ratio	− 0.49	0.2134
Refraction	− 0.68	0.0619
Axial length	0.17	0.6897
Superficial FAZ vs.		
Deep FAZ	0.49	0.2135
fIRL/pIRL ratio Refraction	− 0.55 − 0.65	0.1621 0.0821
Axial length	0.46	0.2554
Deep FAZ vs.		
fIRL/pIRL ratio	− 0.71	0.0496*
Refraction	− 0.54	0.1641
Axial length	0.41	0.3069
fIRL/pIRL ratio vs.		
Refraction	0.74	0.0378*
Axial length	− 0.76	0.0280*
Refraction vs.		
Axial length	− 0.63	0.0965

*BCVA* best-corrected visual acuity, *FAZ* foveal avascular zone, *fIRL* foveal inner retinal layer, *pIRL* parafoveal inner retinal layer

*P < 0.05

## Discussion

In the present study, we confirmed the structural characteristics of the nanophthalmic macula and quantified the FAZ size and the IRL thickness for both the fovea and parafovea using OCTA and SD-OCT. Moreover, we found a significant correlation between the BCVA and the deep FAZ size. In addition, the fIRL/pIRL ratio, an indicator of hypoplasia, was significantly correlated with the refractive error, axial length, and deep FAZ size. However, the fIRL/pIRL ratio was not significantly correlated with either the BCVA or superficial FAZ size.

The measured FAZ sizes were smaller than that observed in normal adult eyes (0.05–1.05 mm^2^) without distinction between vascular layers [13]. Furthermore, the average fIRL/pIRL ratio was 0.46 ± 0.41 with a range from 0.06 to 1.10, which was significantly greater than the average measure for normal adult eyes (0.05, range: 0.00–0.20) [11]. This result indicates that the fIRL thickness is larger in nanophthalmic eyes, which might in turn cause an undeveloped FAZ.

Foveal hypoplasia can be found in patients with one of several other eye disorders such as albinism, PAX6 gene mutations with aniridia, isolated foveal hypoplasia, achromatopsia [14], retinopathy of prematurity [15], and Stickler syndrome [9]. Studies investigating these disorders suggested that OCT was the ideal method to establish the structural grading system of foveal hypoplasia [14]. Furthermore, Matsushita et al. investigated patients with foveal hypoplasia due to Stickler syndrome using OCT and OCTA, in addition to calculating the fIRL/pIRL ratio [9]. Their results appeared to be similar to those found in the present study in terms of the relatively low-grade foveal hypoplasia (mostly Grade 1 or 2), the persistence of the IRL, and the preserved visual acuity in some patients. Therefore, the presence of foveal morphological anomalies is not always predictive of visual acuity impairments.

The fovea is considered a specialized and critical region of the retina because it contains the highest cone photoreceptor density, which enables high visual acuity and color vision [16]. The development of the foveal pit begins at fetal week 25 and the excavation is accomplished between months 15 to 45 after birth [10, 17]. During the foveal developmental process, the foveal pit is created by bidirectional movements of the retinal neuronal cells. The inner retinal cells are relocated outside the foveal pit, whereas cone photoreceptor cells move inward to increase their concentration within the foveal pit [18]. However, those inner and outer retinal layer changes occur separately at different time points. Molecular analysis of the macular region indicates that at fetal week eight, axon guidance molecules act initially to repel axons and subsequently blood vessels to form FAZ [19, 20], which is then followed by foveal pit formation beginning at fetal week 25 [21]. In contrast, outer retinal development, such as cone elongation and packing, mainly occurs postnatally [10]. In the foveal outer segment, the cone density continues to rise until a threefold increase is reached approximately 3.8 years after birth; however, the cause of this increase is not yet known [22].

Furthermore, the relationship between abnormal FAZs (either in superficial or deep capillary plexuses) and visual acuity is not clear. Interestingly, eyes with smaller FAZs in deep layers had better visual acuity despite also having higher fIRL/pIRL ratios compared with other eyes. Previous reports showed that diminished macular vasculature was associated with structural and functional damage in glaucoma [23] and that the vessel density in superficial layers was greater than in deep layers [7]. These findings contradict the present results and do not explain why smaller FAZs in the deep retinal layers are associated with better visual acuity. Therefore, it remains unclear whether morphological abnormalities arising from nanophthalmos lead to visual dysfunctions. In subjects with nanophthalmos, the eyeball cannot fully develop during the embryonic stage, resulting in macular hypoplasia [24]. Since visual acuity reaches maturity long after birth, it is possible that appropriate therapeutic approaches can help prevent visual impairments in nanophthalmos cases while the outer segment of the retina is not yet fully developed. Results from the study of other conditions that also produce foveal hypoplasia such as albinism [25], retinopathy of prematurity [15], and Stickler syndrome [9] also support the present findings indicating that anatomical abnormalities do not always correlate with visual acuity.

Some limitations of the present study should be addressed. First, the sample size was notably small. Accumulating additional OCTA and OCT data might help to determine if a significant association exists between the foveal structure, vessel plexus, and visual outcomes. Second, the automated measurement of the FAZ was not without issues as in some cases it appeared to be inaccurate. In particular, for undeveloped FAZ cases, we found that it tended to overestimated linings, which then require manual adjustments with Photoshop. In addition, there is the possibility of segmentation errors. The segmentation of the superficial capillary plexus using OCTA images is determined as a slab starting 3 µm below the internal limiting membrane and ending 15 µm under the inner plexiform layer [26]. Since the IRL thickness differed for each participant, this may have led to imprecise FAZ measurements.

## Conclusions

We report here OCTA and SD-OCT features for five nanophthalmos cases. We identified abnormal FAZs in the deep and superficial retinal layers, in addition to underdeveloped retinal microstructures. However, our findings did not show a clear relationship between these structural changes and visual function, even though the deep FAZ size was correlated with the BCVA. Further studies with larger sample sizes will be necessary to develop a better understanding of how both variables are linked.
